# Clinical courses and outcomes of COVID-19 associated pulmonary aspergillosis in 168 patients with the SARS-CoV-2 omicron variant

**DOI:** 10.1186/s12879-023-08971-w

**Published:** 2024-01-23

**Authors:** Yixuan Wang, Yan Yao, Qingfeng Zhang, Hao Chen, Yang He, Ke Hu

**Affiliations:** 1 Department of Respiratory and Critical Care Medicine, Renmin Hospital of Wuhan University, Wuhan, 430060 China; 2 Department of Pharmacy, Renmin Hospital of Wuhan University, Wuhan, 430060 China

**Keywords:** COVID-19, CAPA, SARS-CoV-2, Omicron variant, Aspergillosis, NLR, Mortality

## Abstract

**Purpose:**

We aimed to analyze the clinical features of COVID-19-associated pulmonary aspergillosis (CAPA) during the SARS-CoV-2 Omicron variant pandemic and to reveal the risk factors for CAPA and death.

**Methods:**

A retrospective cohort study was conducted on 168 CAPA patients from December 8, 2022 to January 31, 2023. 168 COVID-19 patients without secondary fungal infection during this period were matched 1:1 using propensity score matching as controls.

**Results:**

The incidence of CAPA was 3.8% (168/4421). Compared with patients without fungal infection, CAPA patients had a higher mortality (43.5% vs. 10.1%, *P* < 0.001). Patients in the death group (*n* = 73) were more likely to be admitted to ICU (91.8% vs. 26.3%, *p* < 0.001), had a shorter ICU length of hospitalization (10 (IQR, 6 ~ 16.5) days vs. 14 (IQR, 8 ~ 37) days, *p* = 0.012). Immunocompromised status (*p* = 0.023), NLR ≥ 5.7 (*p* = 0.004), CRP ≥ 50 mg/L (*p* = 0.043), and the number of antibiotics ≥ 3 (*p* < 0.001) were all risk factors for CAPA; NLR ≥ 5.7 (*p* = 0.009) and the number of antibiotics ≥ 3 (*p* = 0.018) were all independent risk factors for death.

**Conclusions:**

During the Omicron variant pandemic, CAPA increased death and ICU length of hospitalization. The risk factors of CAPA and death obtained from the study can help us further understand the disease characteristics of CAPA and better guide our clinical decision-making.

**Supplementary Information:**

The online version contains supplementary material available at 10.1186/s12879-023-08971-w.

## Introduction

Since 2022, the new coronavirus Omicron variant has significantly increased its transmission and immune escape ability compared to previous variants, and has quickly replaced the Delta variant as the dominant strain in the world [1]. Although the Omicron variant is clinically less virulent than the original strain, not everyone infected is mild or asymptomatic. Therefore, the severity and fatality rate of the Omicron variant should not be underestimated. It had been found that some patients with COVID-19 are prone to secondary bacterial and fungal infections [2, 3]. In previous pandemics, the incidence of secondary infection by other pathogens has varied, with secondary fungal infection rates ranging from 1% [4] to 27% [5]. COVID-19-associated pulmonary aspergillosis (CAPA) is now considered a potentially life-threatening secondary infection in a large number of critically ill COVID-19 patients [6]. In a meta-analysis involving eight retrospective studies and a total of 729 patients with COVID-19, 15.0% (109/729) were diagnosed with CAPA, with incidence ranging between 3.3% and 34.4% [7]. Viral damage to the bronchial mucosa and alveolar damage, combined with increased lung epithelium and vascular permeability, may create favorable conditions for aspergillus invasion [8]. In addition, patients with CAPA are often older, have a history of COPD, and are treated with long-term corticosteroids [7]. Increased demand for mechanical ventilation, various immunosuppressive therapies, and multiple organ dysfunction during treatment are all related to the occurrence or death of CAPA [9].

So far, there are very few reports on the infection rate, risk factors, and impact on prognosis of CAPA after Omicron variant infection, and whether the results are the same as those of the previous variant strains is unknown, which should arouse our high attention. Therefore, we compared the clinical characteristics and outcomes of pulmonary aspergillosis in patients with Omicron infection, hoping to facilitate early stratification of COVID-19 patients at high risk for CAPA.

## Methods

### Study design and patients

**Fig. 1:**
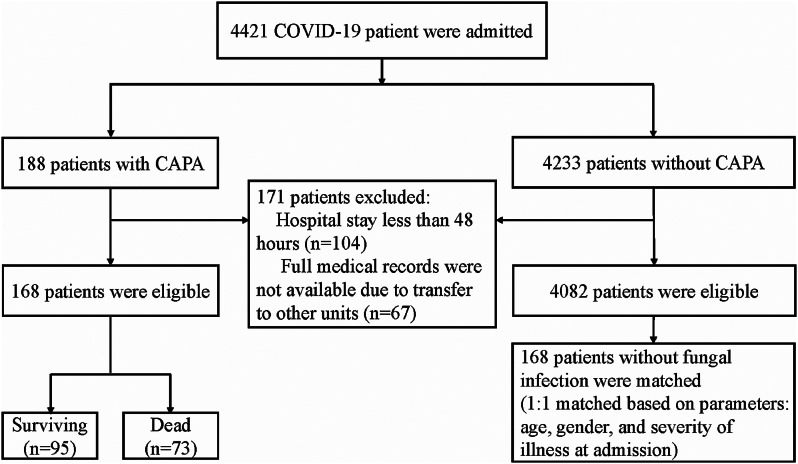
Study flowchart. Abbreviation: CAPA, COVID-19 associated pulmonary aspergillosis

This was a retrospective study. As shown in Fig. 1, between December 8, 2022, and January 31, 2023, a total of 4421 COVID-19 patients were admitted to Renmin Hospital of Wuhan University, China, during the Omicron variant epidemic. Among them, we retrospectively gathered data from 168 patients who met the CAPA diagnostic criteria during the same timeframe. To ensure comparability, the propensity score matching method [10] was employed to match 168 patients without secondary fungal infections in a 1:1 ratio. Specifically, the propensity score of each patient was calculated based on covariates such as gender, age, and severity of illness on admission, and a similar case and control group with balanced characteristics were created.

Inclusion criteria were as follows: Adults (over 18 years of age) with real-time reverse transcription polymerase chain reaction (RT-PCR) testing confirmed severe acute respiratory syndrome coronavirus 2 (SARS-CoV-2) infection. The exclusion criterion was as follows: (a) Hospital stay less than 48 h; (b)Full medical records were not available due to transfer to other units.

The diagnosis of CAPA is based on 2020 ECMM/ISHAM consensus criteria [6]. According to the criteria, patients were categorized as proven CAPA, probable CAPA, possible CAPA or no evidence for CAPA.

Due to the nature of the study as a retrospective data review, the need for individual patient informed consent was waived with the consent of the Ethics Committee of Renmin Hospital of Wuhan University.

### Data acquisition

Demographic information (age, gender), clinical characteristics (medical history, underlying comorbidities, signs and symptoms), laboratory results, treatment and outcomes (length of hospital stay, intensive care unit (ICU) admission and mortality) were extracted from the electronic medical record system of Renmin Hospital of Wuhan University.

### Definition of primary variates and outcomes

The definition of Immunocompromised status refers to a patient who has been receiving steroid or immunosuppressive therapy before admission. COVID-19 severity: Mild: mild symptoms (e.g., low-grade fever, mild respiratory symptoms like cough and sore throat) without pneumonia or hypoxia. Moderate: sustained high fever (> 3 days) with cough, dyspnea, radiographic evidence of typical COVID-19 pneumonia, respiratory rate < 30 breaths/min, and resting oxygen saturation ≥ 94%. Severe: meets any of the following criteria: (1) Respiratory rate ≥ 30 breaths/min; (2) Resting oxygen saturation < 94% on room air; (3) PaO2/FiO2 ratio < 300 mmHg; (4) Progressive worsening of clinical symptoms with > 50% increase in lung lesion progression within 24–48 h based on radiographic findings. Critical: meets any of the following conditions: (1) Respiratory failure requiring mechanical ventilation; (2) Shock development; (3) Multiple organ failure necessitating intensive care unit monitoring and treatment. Neutrophil-lymphocyte ratio (NLR) was calculated by dividing the absolute neutrophil count by the absolute lymphocyte count for the same automated complete blood count sample.

### Statistical analysis

For the purpose of this study, a descriptive analysis of various clinical information and laboratory tests was performed. The normality test was carried out by the Kolmogorov-Smirnov method, and the measurement data that met the normal distribution were expressed as mean ± standard deviation, and the comparison between groups was performed by T test; non-normally distributed measures were expressed as median (interquartile range (IQR)), and were compared with a Mann-Whitney test. Categorical variables were expressed as absolute numbers or percentages and using the exact Chi-square (χ2) and Fisher’s exact test. The Kaplan-Meier cumulative event curves for 28-day all-cause death in the two groups were constructed, and the difference between the two groups was compared using the log rank test. Multivariate logistic regression and multivariate Cox regression models were utilized to evaluate the risk factors with CAPA and death, respectively. The results are expressed as odds ratios (OR) or hazard ratios (HR) with corresponding 95% confidence intervals (CI). All statistical analyses were performed in SPSS version 23.0 (IBM, New York, USA). Figures were generated using GraphPad Prism (GraphPad Software, San Diego, Canada) version 9.0. P values < 0.05 were considered statistically significant.

## Results

### Clinical characteristics and laboratory results of CAPA group and non-fungal infection group

A total of 168 CAPA patients and matched 168 COVID-19 patients without fungal infection during the study period were included in the final analysis. Table 1 summarized the clinical characteristics of the two arms. The CAPA group were more likely to smoke (23.8% vs. 11.9%, *p* = 0.007). In terms of comorbidities, we noted that both groups were dominated by comorbid hypertension, but there was no statistical difference in the prevalence of hypertension between the two groups (*p* = 0.230). However, the proportion of patients with combined coronary heart disease (25.0% vs. 13.1%, *p* = 0.008) and chronic obstructive pulmonary disease (13.1% vs. 5.4%, *p* = 0.022) was significantly higher in the CAPA group compared with the group without fungal infection. Except for the differences in shortness of breath and fatigue, there were no significant differences in other first symptoms and vital signs between the two groups (all *p* > 0.05). With the progression of the disease, the proportion of severe/critical cases in the CAPA group was significantly higher than that in the group without fungal infection (*p* < 0.001).

**Table 1:** Comparison of demographic, comorbidity, and clinical characteristics of CAPA group and non-fungal infection group

Variables	CAPA (*n* = 168)	Non-fungal infection (*n* = 168)	P
Male, n (%)	124 (73.8)	118 (70.2)	0.544
Age, years	74.5 (62.0 ~ 83.0)	71.0 (59.0 ~ 82.0)	0.367
Smoking history, n (%)	40 (23.8)	20 (11.9)	**0.007**
History of alcohol consumption, n (%)	20 (11.9)	17 (10.1)	0.728
**Comorbidity, n (%)**			
Hypertension	91 (54.2)	79 (47.0)	0.230
Diabetes	46 (27.4)	39 (23.2)	0.452
Coronary artery disease	42 (25.0)	22 (13.1)	**0.008**
Chronic obstructive pulmonary disease	22 (13.1)	9 (5.4)	**0.022**
Cerebrovascular disease	24 (14.3)	19 (11.3)	0.514
Malignancy	30 (17.9)	20 (11.9)	0.167
Chronic renal insufficiency	21 (12.5)	10 (6.0)	0.058
HIV infection	2 (1.2)	0 (0)	0.499
Immunocompromised status	32 (19.0)	24 (14.3)	0.306
**First symptoms**			
Fever (temperature ≥ 37.0℃), n (%)	147 (87.5)	133 (79.2)	0.056
Maximum temperature, ℃, median (IOR)	38.8 (38.2 ~ 39.5)	38.9 (38.0 ~ 39.2)	0.178
Cough, n (%)	135 (80.4)	145 (86.3)	0.187
Expectoration, n (%)	119 (70.8)	118 (70.2)	1.000
Chest tightness, n (%)	69 (41.1)	51 (30.4)	0.053
Shortness of breath, n (%)	80 (47.6)	38 (22.6)	**< 0.001**
Fatigue, n (%)	91 (54.2)	69 (41.1)	**0.022**
Myalgia, n (%)	5 (3.0)	28 (16.7)	**< 0.001**
Nasal congestion, runny nose, n (%)	12 (7.14)	10 (6.0)	0.826
Nausea, vomiting, n (%)	13 (7.7)	14 (8.3)	1.000
Abdominal pain, diarrhea, n (%)	19 (11.3)	12 (7.1)	0.258
**Vital signs on admission**			
Systolic blood pressure, mmHg	128.9 ± 22.6	128.4 ± 18.5	0.826
Diastolic blood pressure, mmHg	74.1 ± 12.7	73.7 ± 10.6	0.768
Heart rate, beats/min	88.6 ± 19.1	87.6 ± 17.2	0.593
Respiratory rate, breaths/min	20.0 (18.0 ~ 22.0)	20.0 (19.0 ~ 20.0)	0.806
Body temperature, ℃	36.5 (36.3 ~ 36.8)	36.5 (36.3 ~ 36.8)	0.771
**Severity of illness at admission, n (%)**			
Mild and moderate	111 (66.1)	120 (71.4)	0.346
Severe and critical	57 (33.9)	48 (28.6)	
**Severity of illness at worst, n (%)**			
Mild and moderate	30 (17.9)	97 (57.7)	**< 0.001**
Severe and critical	138 (82.1)	71 (42.3)	

Bolded terms represent subheadings. Bold values represent p < 0.05, indicating statistical significance

Abbreviations: CAPA, COVID-19-associated pulmonary aspergillosis; HIV, human immunodeficiency virus

The laboratory results of the two groups are presented in Supplementary Table 1. The CAPA group exhibited lower SaO_2_, PaO_2_, lymphocyte count, hemoglobin, albumin, and higher lactate, white blood cell count, neutrophil count, NLR, CRP, LDH, D-dimer and NT-proBNP compared to the group without fungal infection. And these differences were more significant with disease progression. Immune function tests on admission in both groups suggested that CAPA patients had higher levels of IL-6 and IL-10, and lower levels of CD3, CD4, CD8, and CD16 + 56.

**Fig. 2:**
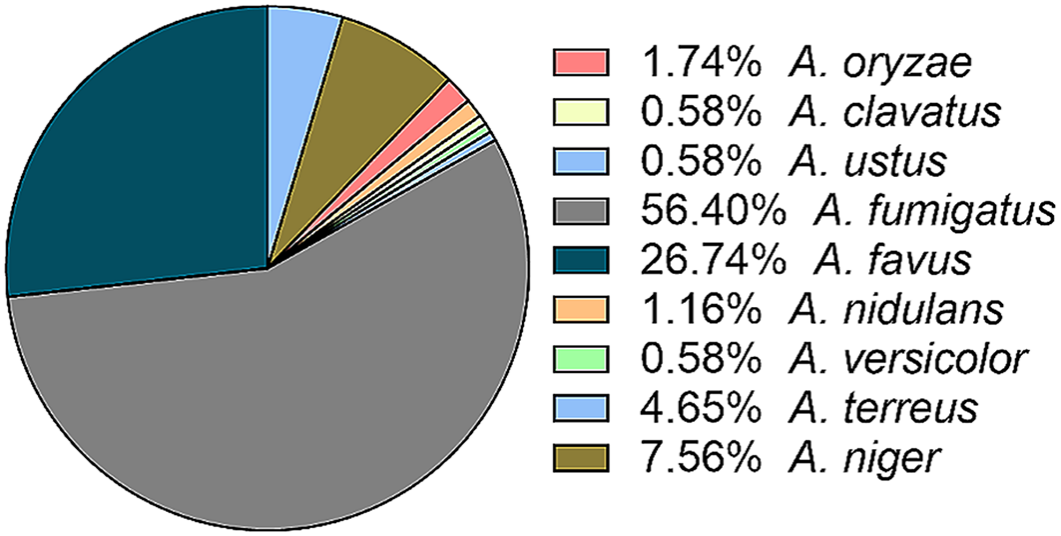
Details of Aspergillus isolates within the CAPA group

The Aspergillus isolates within the CAPA group are delineated in Fig. 2. Notably, pathogenic species encompassed *A. fumigatus* in 97 cases (56.4%), *A. flavus* in 46 cases (26.74%), *A. niger* in 13 cases (7.56%), and *A. terreus* in 8 cases (4.65%). Additionally, a minor fraction comprised other Aspergillus species. It is noteworthy that 4 cases (2.38%) detected multiple strains of Aspergillus.

### Treatments and outcomes of CAPA group and non-fungal infection group

**Fig. 3:**
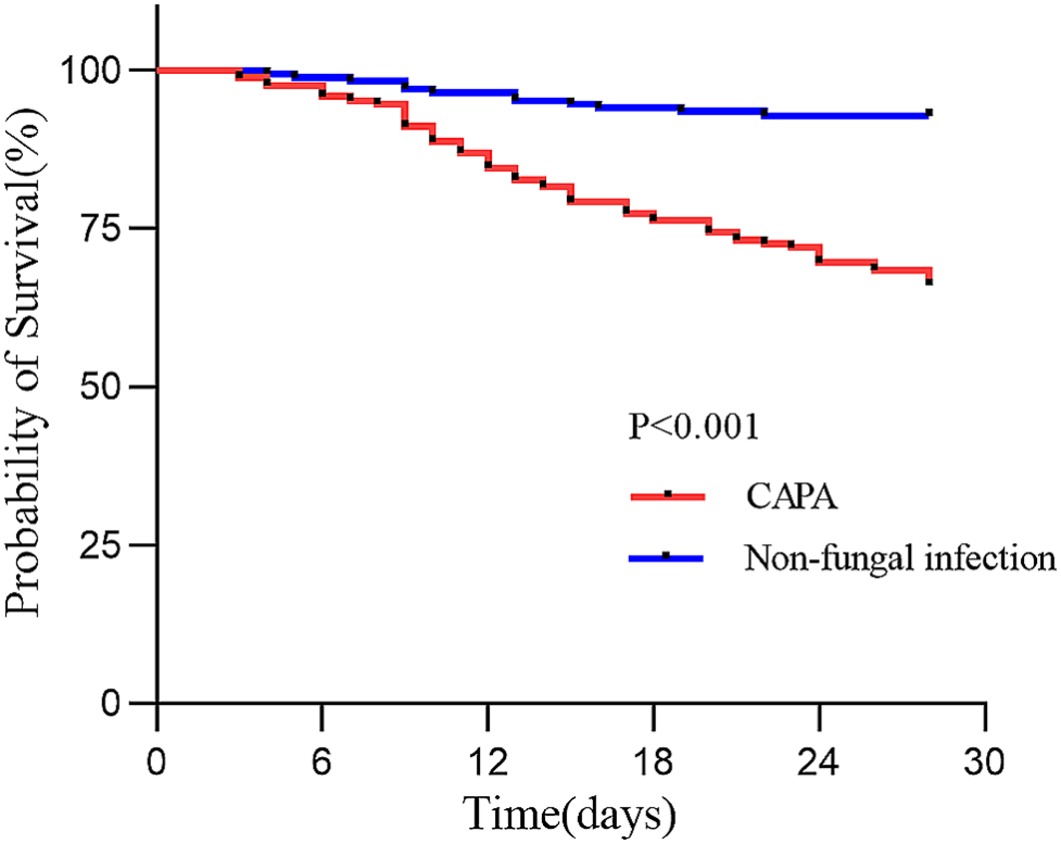
Kaplan-Meier curves of 28-day mortality in CAPA group and non-fungal infection group. Abbreviation: CAPA, COVID-19 associated pulmonary aspergillosis

Compared with the non-fungal infection group, the CAPA group had more patients admitted to the ICU before diagnosis (50.6% vs. 13.1%), received more types of antibiotics (3 (IQR, 2 ~ 4) vs. 1 (IQR, 1 ~ 2), *p* < 0.001), had more invasive operations, and higher mortality (43.5% vs. 10.1%, *p* < 0.001). (Table 2). At the same time, the Kaplan-Meier curve showed that the 28-day mortality rate of the CAPA group was also significantly higher than that of the non-fungal infection group (log rank *p* < 0.001) (Fig. 3). In addition, 137 (81.5%) individuals in the CAPA group received antifungal treatment, with 113 individuals using mould-active azoles (voriconazole, *n* = 98; itraconazole, *n* = 18), 43 individuals using amphotericin B, and 19 receiving a combination of both. In multivariate logistic regression analysis, Immunocompromised status (OR:2.220, 95% CI:1.116 ~ 4.414), NLR ≥ 5.7 (OR:1.726, 95% CI:1.017 ~ 2.930), CRP ≥ 50 mg/L (OR:2.182, 95% CI:1.279 ~ 3.721) and the number of antibiotics ≥ 3 (OR:3.926, 95% CI:2.243 ~ 6.869) were risk factors for CAPA (Table 3).

**Table 2:** Comparison of interventions between the CAPA group and non-fungal infection group

Variables	CAPA** (*n* = 168)	Non-fungal infection (*n* = 168)	P
ICU admission, n (%)	85 (50.6)	22 (13.1)	**< 0.001**
ICU length of hospitalization, days	2 (1 ~ 7)	6.0 (3.0 ~ 8.5)	**0.006**
Mortality, n	73 (43.5)	17 (10.1)	**< 0.001**
The length of hospital stays, days	9 (6 ~ 14)	9 (7 ~ 16)	0.179
**Number of antibiotics, n**	3 (2 ~ 4)	1 (1 ~ 2)	**< 0.001**
Meropenem, n (%)	52 (31.0)	21 (12.5)	**< 0.001**
Vancomycin, n (%)	15 (8.9)	4 (2.4)	**0.016**
Teicoplanin, n (%)	21 (12.5)	9 (5.4)	**0.034**
Imipenem, n (%)	45 (26.8)	17 (10.1)	**< 0.001**
Etimicin, n (%)	10 (6.0)	1 (0.6)	**0.011**
Fluoroquinolones, n (%)	101 (60.1)	113 (67.3)	0.212
Polymyxin B, n (%)	8 (4.8)	1 (0.6)	**0.037**
Linezolid, n (%)	13 (7.7)	4 (2.4)	**0.044**
Cephalosporin, n (%)	132 (78.6)	76 (45.2)	**< 0.001**
Tigecycline, n (%)	4 (2.4)	0 (0)	0.123
Duration of antibiotic therapy, days	7 (4 ~ 12)	11.3 ± 11.0	**0.005**
**Anti-fungal therapy, n**	137 (81.5)	N/A	N/A
Mould-active azole, n (%)	113 (67.3)	N/A	N/A
Amphotericin B, n (%)	43 (26.0)	N/A	N/A
Ganciclovir, n (%)	10 (6.0)	12 (7.1)	0.826
Ribavirin, n (%)	0 (0)	0 (0)	1.000
Amubarvimab, n (%)	8 (4.8)	25 (14.9)	**0.003**
Tocilizumab, n (%)	9 (5.4)	10 (6.0)	1.000
Antiviral therapy**#**, n (%)	88 (52.4)	74 (44.0)	0.156
Traditional Chinese Medicine, n (%)	15 (8.9)	14 (8.3)	1.000
Immunoglobulin, n (%)	70 (41.7)	46 (27.4)	**0.008**
Thymosin, n (%)	14 (8.3)	6 (3.6)	0.105
Glucocorticoid, n (%)	100 (59.5)	144 (85.7)	**< 0.001**
Duration of glucocorticoid therapy, days	7 (4 ~ 11)	8 (6 ~ 10)	0.188
Cumulative glucocorticoid dose*, mg	1577 (950 ~ 2358)	1400 (1100 ~ 2000)	0.478
Oxygen therapy, n (%)	156 (92.9)	99 (58.9)	**< 0.001**
High-flow oxygen therapy, n (%)	68 (40.5)	25 (14.9)	**< 0.001**
Non-invasive mechanical ventilation, n (%)	18 (10.7)	13 (7.7)	0.451
Invasive mechanical ventilation, n (%)	60 (35.7)	18 (10.7)	**< 0.001**
ECMO, n (%)	3 (1.8)	1 (0.6)	0.623
**Invasive operation, n (%)**	116 (69.1)	45 (26.8)	**< 0.001**
Indwelling urinary catheterization, n (%)	98 (58.3)	25 (14.9)	**< 0.001**
Central venous catheterization, n (%)	72 (42.9)	34 (20.2)	**< 0.001**
Puncture and drainage, n (%)	18 (10.7)	6 (3.6)	**0.018**
Tracheal intubation, n (%)	44 (26.2)	16 (9.5)	**< 0.001**
Tracheotomy, n (%)	12 (7.1)	6 (3.6)	0.225
Fiberoptic bronchoscopy, n (%)	23 (13.7)	14 (8.3)	0.163
CRRT, n (%)	11 (6.5)	4 (2.4)	0.110
Artificial liver treatment, n (%)	0 (0)	0 (0)	1.000

Bolded terms represent subheadings. Bold values represent p < 0.05, indicating statistical significance

Abbreviations: CAPA, COVID-19-associated pulmonary aspergillosis; ICU, intensive care unit; N/A, Not Applicable; ECMO, extracorporeal membrane oxygenation; CRRT, continuous renal replacement therapy. #Antiviral therapy including Naimatrelvir/Ritonavir and Azvudine. *Hydrocortisone dose was taken as reference. **The data were collected at the time of CAPA diagnosis, except for the data on “Anti-fungal therapy” and “Mortality”

**Table 3:** Multivariate logistic regression analysis of risk factors for CAPA

Variables		P	OR	95% CI
Variables in equation	Immunocompromised status	**0.023**	2.220	1.116 ~ 4.414
	CRP ≥ 50 mg/L	**0.004**	2.182	1.279 ~ 3.721
	NLR ≥ 5.7	**0.043**	1.726	1.017 ~ 2.930
	Number of antibiotics ≥ 3	**< 0.001**	3.926	2.243 ~ 6.869
Variables not in equation	Age	0.751		
	Sex	0.648		
	Diabetes	0.565		
	Chronic obstructive pulmonary disease	0.139		
	Tocilizumab	0.285		
	Cumulative glucocorticoid dose* ≥1500 mg	0.077		

Bold values represent p < 0.05, indicating statistical significance

Abbreviations: CAPA, COVID-19-associated pulmonary aspergillosis; CRP, C-reactive protein; NLR, neutrophil-lymphocyte ratio; OR, odds ratio; CI, confidence interval; *Hydrocortisone dose was taken as reference

### Clinical characteristics and laboratory results of survival group and death group with CAPA

Further delving into the causes of high mortality of CAPA, we divided the CAPA patients into survival group (*n* = 95) and death group (*n* = 73) and compared the demographic and clinical characteristics between the two groups (Supplemental Table 2). Both groups were predominantly male, but the proportion of males in the death group was significantly higher than that in the survivor group ([M: F], 60:13 vs. 64:31, *p* = 0.035). There were no significant differences in age, first symptoms, vital signs on admission, or comorbidities between the two groups. However, the length of hospital stay in the death group was longer than that in the survival group (23 (IQR, 15.0 ~ 35.5) days vs. 17 (IQR, 10 ~ 28) days, *p* = 0.001).

The laboratory results are shown in Table 4, compared to the survival group, the death group exhibited lower SaO_2_, PaO_2_, lymphocyte count, platelet count, and higher lactate levels, neutrophil count, NLR, CRP, AST, urea, creatinine, LDH, D-dimer, PCT, CKMB, ultratroponin I, MYO, NT-proBNP at admission, and these differences became more significant as the disease progressed. Examination of inflammatory markers between the two groups revealed that the death group had higher levels of IL-6, IL-10 than the survival group. The immune function test showed that the CD3 count, CD4 count, CD8 count, as well as CD16 + 56 count were significantly lower in the death group than in the survival group.

**Table 4:** Comparison of laboratory Results between survival and death groups in CAPA patients on admission

Variables	Survival group (*n* = 95)	Death group (*n* = 73)	P
SaO_2_ (95 ~ 98%)	96 (94 ~ 98.4)	91 (85 ~ 96)	**0.027**
PaO_2_ (83 ~ 108 mmHg)	81 (70.5 ~ 101)	67 (55.8 ~ 95.3)	**0.003**
Lactic acid (0.36 ~ 1.25 mmol/L)	1.5 (1 ~ 1.8)	1.8 (1.1 ~ 2.5)	**0.014**
WBC count (3.5 ~ 9.5 × 10^9^/L)	6.9 (4.6 ~ 10.0)	8.4 (5.3 ~ 12.3)	0.068
Neutrophil count (1.8 ~ 6.3 × 10^9^/L)	4.7 (2.9 ~ 8.3)	7.9 (4.4 ~ 11.9)	**0.002**
Lymphocyte count (1.1 ~ 3.2 × 10^9^/L)	0.9 (0.5 ~ 1.2)	0.6 (0.4 ~ 0.9)	**0.009**
Lymphocyte count < 1.0 × 10^9^/L, n (%)	56 (59.0)	58 (79.45)	**0.005**
NLR (0.78 ~ 3.53)	5.9 (2.8 ~ 14.6)	10.4 (5.7 ~ 27.0)	**0.001**
Hemoglobin (130 ~ 175 g/L)	117 (101 ~ 132.8)	126 (103 ~ 139)	0.179
Platelet count (125 ~ 350 × 10^9^/L)	188.5 (152.3 ~ 247.8)	160 (134 ~ 197)	**0.004**
CRP (0 ~ 10 mg/L)	45.7 (11.0 ~ 85.7)	60.5 (27.9 ~ 17.0)	**0.018**
Albumin (40 ~ 55 g/L)	34 (31.4 ~ 38.2)	33.9 (31 ~ 37.1)	0.672
ALT (9 ~ 50 U/L)	26 (14 ~ 35)	28 (19 ~ 43)	0.111
AST (15 ~ 40 U/L)	27.5 (18.3 ~ 41.8)	41 (26 ~ 64)	**< 0.001**
ALP (45 ~ 125 U/L)	66 (55.5 ~ 86)	73 (59 ~ 108)	0.073
Bilirubin (0 ~ 23 mmol/L)	10.3 (7.2 ~ 13.9)	11.8 (8.3 ~ 17.9)	0.234
Potassium (3.5 ~ 5.3 mmol/L)	4.0 (3.6 ~ 4.4)	4.03 (3.6 ~ 4.7)	0.421
Sodium (137 ~ 147 mmol/L)	138.5 (135 ~ 141.6)	139 (135.4 ~ 142.1)	0.429
Urea (3.6 ~ 9.5 mmol/L)	6.4 (5.1 ~ 11.2)	10 (6.5 ~ 14.5)	**0.001**
Creatinine (57 ~ 111 µmol/L)	77.5 (54.5 ~ 112.3)	96 (70 ~ 133)	**0.005**
LDH (120 ~ 250 U/L)	295 (227.5 ~ 355.5)	432.5 (313.5 ~ 503)	**< 0.001**
D-dimer (0 ~ 0.55 mg/L)	1.2 (0.7 ~ 3.7)	2.2 (0.7 ~ 9.2)	**0.039**
PT(9 ~ 13 s)	12.3 (11.6 ~ 13.3)	12.4 (11.4 ~ 13.5)	0.962
Fibrinogen (2 ~ 4 g/dL)	4.4 (3.4 ~ 5.5)	4.6 (3.3 ~ 5.8)	0.691
APTT (25 ~ 31.3 s)	30.6 (27 ~ 33.4)	31 (27.6 ~ 33.6)	0.334
PCT (< 0.05 ng/mL)	0.11 (0.06 ~ 0.6)	0.47 (0.15 ~ 1.43)	**0.006**
CKMB (0 ~ 5 ng/mL)	1 (0.3 ~ 1.8)	1.6 (0.8 ~ 3.6)	**0.001**
Ultratroponin I (0 ~ 0.04 ng/mL)	0.03 (0.01 ~ 0.07)	0.06 (0.02 ~ 0.18)	**0.009**
MYO (0 ~ 110 ug/ml)	86.4 (56.5 ~ 166.1)	166.7 (94.1 ~ 435.6)	**< 0.001**
NT-proBNP (0 ~ 125 pg/mL)	462 (199.5 ~ 1345)	755 (277 ~ 2439.5)	**0.032**
IL-2 (≤ 11.4 pg/mL)	2.6 (2.3 ~ 3.05)	2.6 (2.4 ~ 3.4)	0.636
IL-4 (≤ 12.9 pg/mL)	4.9 (4.6 ~ 5.2)	4.8 (4.6 ~ 5.1)	0.333
IL-6 (≤ 20.0 pg/mL)	32 (11 ~ 56.8)	47.3 (22.4 ~ 243.1)	**0.007**
IL-10 (≤ 5.9 pg/mL)	6.1 (4.7 ~ 8.5)	10 (6.7 ~ 17.1)	**< 0.001**
TNF (≤ 5.5 pg/mL)	3.4 (2.9 ~ 5.1)	3.1 (2.7 ~ 4.0)	0.187
Interferon-γ (≤ 17.3 pg/mL)	3 (2.4 ~ 4.2)	2.5 (2.2 ~ 3.9)	0.049
IgG (8.6 ~ 17.4 g/L)	11 (9.3 ~ 11.9)	12.6 (9.2 ~ 15.3)	0.050
IgM (0.3 ~ 2.2 g/L)	0.8 (0.6 ~ 1)	0.7 (0.6 ~ 1.1)	0.965
IgA (1 ~ 4.2 g/L)	2.3 (1.8 ~ 2.6)	1.8 (1.4 ~ 2.5)	0.197
IgE (0 ~ 100 g/L)	81.4 (18.4 ~ 183)	92.7 (18.4 ~ 176)	0.775
C3 (0.7 ~ 1.4 g/L)	0.7 (0.5 ~ 0.9)	0.7 (0.5 ~ 0.9)	0.561
C4 (0.1 ~ 0.4 g/L)	0.2 (0.1 ~ 0.2)	0.2 (0.1 ~ 0.2)	0.916
CD3 (56 ~ 86%)	67.6 (60.7 ~ 73.9)	58.1 (42.3 ~ 65.2)	**< 0.001**
CD3 count (723 ~ 2737 pcs/uL)	450.5 (282.3 ~ 724.5)	201 (123 ~ 275)	**< 0.001**
CD4(33 ~ 58%)	36.9 (32.8 ~ 45.9)	29.1 (21.5 ~ 40.5)	**0.002**
CD4 count (404 ~ 1612 pcs/uL)	254 (149.8 ~ 421.5)	109 (52.5 ~ 175.3)	**< 0.001**
CD8 (19 ~ 38%)	21.6 (16.7 ~ 33.5)	19.6 (13.8 ~ 29.7)	0.146
CD8 count (220 ~ 1129 pcs/uL)	145.5 (97.5 ~ 277.8)	72.5 (41.3 ~ 109.8)	**< 0.001**
CD19(5 ~ 22%)	12.1 (7.1 ~ 17.5)	22.2 (10.6 ~ 32.3)	**0.003**
CD19 count (80 ~ 616 pcs/uL)	79 (36.3 ~ 165)	69 (34.8 ~ 123.8)	0.645
CD16 + 56 (5 ~ 26%)	15.8 (11.8 ~ 20.6)	17.8 (7.8 ~ 27.6)	0.806
CD16 + 56 count (84 ~ 724 pcs/uL)	119 (57.8 ~ 184.5)	49.5 (27.5 ~ 105.5)	**0.002**

Bold values represent p < 0.05, indicating statistical significance

Abbreviations: CAPA, COVID-19-associated pulmonary aspergillosis; SaO_2_, oxygen saturation; PaO_2_, partial pressure of oxygen; WBC, white blood cell; NLR, neutrophil-lymphocyte ratio; CRP, C-reactive protein; ALT, alanine aminotransferase; AST, aspartate aminotransferase; ALP, alkaline phosphatase; LDH, lactate dehydrogenase; PT, prothrombin time; APTT, activated partial thromboplastin time; PCT, procalcitonin; CKMB, creatine kinase isoenzyme MB; MYO, myoglobin; NT-proBNP, N-terminal pro-B-type natriuretic peptide; IL, interleukin; TNF, tumor necrosis factor; IgG, immunoglobulin G; IgM, immunoglobulin M; IgA, immunoglobulin A; IgE, immunoglobulin E; C3, complement 3; C4, complement 4; CD, cluster of Differentiation

### Treatments and multivariate analysis of survival group and death group with CAPA

The treatments are presented in Table 5, and compared with the survival group, patients in the death group were more likely to be admitted to ICU (91.8% vs. 26.3%, *p* < 0.001), had a shorter ICU length of hospitalization (10 (IQR, 6.0 ~ 16.5) days vs. 14 (IQR, 8 ~ 37) days, *p* = 0.012), and broad-spectrum antibiotic species were more heterogeneous (4 (IQR, 3 ~ 5) vs. 2 (IQR, 2 ~ 4), *p* < 0.001), especially meropenem, vancomycin, teicoplanin, imipenem, polymyxin B, and linezolid, which were used in a higher proportion of patients than in the survival group. At the same time, we also found that more people treated with Traditional Chinese Medicine, immunoglobulin, glucocorticoids, high flow oxygen inhalation, invasive ventilation and ECMO in the death group than in the survival group, and cumulative glucocorticoid dose was also higher in the death group. The proportion of patients who underwent these invasive procedures including indwelling catheterization, intravenous catheterization, endotracheal intubation, tracheotomy, fiberoptic bronchoscopy, and CRRT was higher in the death group than in the survival group.

**Table 5:** Comparison of interventions between survival group and death group in patients with CAPA

Variables	Survival group (*n* = 95)	Death group (*n* = 73)	P
ICU admission, n (%)	25 (26.3)	67 (91.8)	**< 0.001**
ICU length of hospitalization, days	14 (8 ~ 37)	10 (6 ~ 16.5)	**0.012**
Total length of hospital stays, day	23 (15.0 ~ 35.5)	17 (10 ~ 28)	**0.001**
**Number of antibiotics, n**	2 (2 ~ 4)	4 (3 ~ 5)	**< 0.001**
Meropenem, n (%)	36 (37.9)	43 (58.9)	**0.008**
Vancomycin, n (%)	8 (8.4)	15 (20.6)	**0.040**
Teicoplanin, n (%)	15 (15.8)	22 (30.1)	**0.038**
Imipenem, n (%)	21 (22.1)	43 (58.9)	**< 0.001**
Etimicin, n (%)	9 (9.5)	3 (4.1)	0.234
Fluoroquinolones, n (%)	62 (65.3)	43 (58.9)	0.425
Polymyxin B, n (%)	7 (7.4)	23 (31.5)	**< 0.001**
Linezolid, n (%)	4 (4.2)	20 (27.4)	**< 0.001**
Cephalosporin, n (%)	73 (76.8)	59 (80.8)	0.574
Tigecycline, n (%)	2 (2.1)	4 (5.5)	0.405
Duration of antibiotic therapy, days	20 (13 ~ 29.5)	17 (9 ~ 26)	0.083
Ganciclovir, n (%)	5 (5.3)	7 (9.6)	0.368
Ribavirin, n (%)	0 (0)	0 (0)	-
Amubarvimab, n (%)	6 (6.3)	4 (5.5)	1.000
Tocilizumab, n (%)	8 (8.4)	5 (6.9)	0.778
Antiviral therapy#, n (%)	50 (52.6)	42 (57.5)	0.537
Traditional Chinese Medicine, n (%)	16 (16.8)	25 (34.2)	**0.011**
**Immunoglobulin, n (%)**	47 (49.5)	55 (75.3)	**< 0.001**
Thymosin, n (%)	13 (13.7)	6 (8.2)	0.330
Glucocorticoid, n (%)	70 (73.7)	65 (89.0)	**0.018**
Duration of glucocorticoid therapy, days	10 (6.3 ~ 16.8)	9 (6 ~ 16)	0.663
Cumulative glucocorticoid dose *, mg	1800 (1276 ~ 3037.5)	2600 (1500 ~ 4000)	**0.027**
Oxygen therapy, n (%)	83 (87.4)	73 (100)	**0.001**
High-flow oxygen therapy, n (%)	30 (31.6)	47 (64.4)	**< 0.001**
Non-invasive mechanical ventilation, n (%)	7 (7.4)	11 (15.1)	0.134
Invasive mechanical ventilation, n (%)	12 (12.6)	60 (82.2)	**< 0.001**
ECMO, n (%)	1 (1.1)	9 (12.3)	**0.003**
**Invasive operation, n (%)**	62 (65.3)	73 (100)	**< 0.001**
Indwelling urinary catheterization, n (%)	37 (39.0)	66 (90.4)	**< 0.001**
Central venous catheterization, n (%)	42 (44.2)	69 (94.5)	**< 0.001**
Puncture and drainage, n (%)	15 (15.8)	9 (12.3)	0.658
Tracheal intubation, n (%)	11 (11.6)	59 (80.8)	**< 0.001**
Tracheotomy, n (%)	5 (5.3)	22 (30.1)	**< 0.001**
Fiberoptic bronchoscopy, n (%)	22 (23.1)	37 (50.7)	**< 0.001**
CRRT, n (%)	3 (3.2)	28 (38.4)	**< 0.001**
Artificial liver treatment, n (%)	1 (1.1)	0 (0)	1.000

Bolded terms represent subheadings. Bold values represent p < 0.05, indicating statistical significance

Abbreviations: CAPA, COVID-19-associated pulmonary aspergillosis; ICU, intensive care unit; ECMO, extracorporeal membrane oxygenation; CRRT, continuous renal replacement therapy. **#**Antiviral therapy including Naimatrelvir/Ritonavir and Azvudine; *Hydrocortisone dose was taken as reference

Multivariable Cox regression models were performed for 28-day mortality in CAPA patients. We explored some of the same variables as risk factors for CAPA (Table 6), and the final results suggested that NLR ≥ 5.7 (HR = 2.535, 95%CI: 1.257 ~ 5.112) and the number of antibiotics ≥ 3 (HR = 2.517, 95%CI: 1.170 ~ 5.413) were all independent risk factors for death from CAPA.

**Table 6:** Multivariable Cox regression analysis of 28-day mortality in CAPA patients

Variables		P	HR	95% CI
Variables in equation	NLR ≥ 5.7	**0.009**	2.535	1.257 ~ 5.112
	Number of antibiotics ≥ 3	**0.018**	2.517	1.170 ~ 5.413
Variables not in equation	Age	0.731		
	Sex	0.218		
	Diabetes	0.243		
	Chronic obstructive pulmonary disease	0.421		
	Immunocompromised status	0.100		
	CRP ≥ 50 mg/L	0.182		
	Tocilizumab	0.911		
	Cumulative glucocorticoid dose* ≥1500 mg	0.981		

Bold values represent p < 0.05, indicating statistical significance

Abbreviations: CAPA, COVID-19-associated pulmonary aspergillosis; NLR, neutrophil-lymphocyte ratio; CRP, C-reactive protein; HR, hazard ratio; CI, confidence interval; *Hydrocortisone dose was taken as reference

## Discussion

In this study, we revealed the clinical characteristics of CAPA patients in the context of the Omicron strain pandemic. Immunocompromised status, NLR ≥ 5.7, CRP ≥ 50 mg/L, and the number of antibiotics ≥ 3 was all identified as risk factors for CAPA. Death group of patients with CAPA often received more complicated treatments, NLR ≥ 5.7 and the number of antibiotics ≥ 3 were independent risk factors for death.

Previous studies have reported that secondary fungal infections may be associated with a significantly increased risk of death in critically ill patients infected with SARS-CoV-2. Notably, COVID-19 associated candidiasis (CAC) appears to be less prevalent in this population, whereas CAPA exhibits a higher incidence, potentially resulting in a mortality rate three times greater [11]. These were all concluded from early variant virus strains of the novel coronavirus. Our study discussed the body response (clinical features, laboratory tests) and clinical outcomes in CAPA patients to recent Omicron strain infection. The results showed that CAPA patients had a 43.5% mortality rate, representing about 4-fold increase in mortality compared to the 10.1% in patients without fungal infection. This seems to reflect the harmfulness of CAPA from another angle, and also echoes the previous non-omicron variants research results [12]. In addition, CAPA during the SARS-CoV-2 omicron variant pandemic prolonged the patient’s hospital stay, worsened the severity of the condition, and required more ventilatory and hemodynamic support. These conditions were not apparent during the first wave of the COVID-19 pandemic [7].

The previous association between mortality and gender is also corroborated here, with more deaths in men than in women. These differences may follow gender differences in immune responses and cardiovascular comorbidities [13]. Severe lymphopenia and lymphocyte dysfunction in COVID-19 may affect the development of fungal co-infection [13], and severe impairment of cellular immunity may also increase susceptibility to secondary fungal infection [14]. Focusing on the disease changes caused by Omicron variants, we also observed decreased lymphocytes and impaired cellular immune function in CAPA patients, especially in the death group. Therefore, heightened attention should be given to CAPA patients exhibiting significant elevation in various cytokines and compromised cellular immune function. More interestingly, in our study, it was observed that Immunocompromised status and CRP ≥ 50 mg/L were risk factors for CAPA. Previous studies have also shown that CRP > 10 mg/dL is also related to COVID-19 hyperinflammatory syndrome and subsequent cytokine storm, often indicating more severe outcomes. The maximum CRP value and CRP increase rate can also predict COVID-19 severity [15]. Previous studies have heterogeneity and small sample sizes, so our study further illustrates the close relationship between the two to a certain extent.

Glucocorticoid, sepsis, advanced age and mechanical ventilation have been identified as independent risk factors for mortality in COVID-19 patients in a previous study [16, 17], but our study revealed that the number of antibiotics ≥ 3 and NLR ≥ 5.7 these new risk factors for death. Previous study has suggested that frequency and diversity of antibiotic exposure may be associated with COVID-19 severity and related hospitalizations [18], and our study appears to confirm this. It is known that direct effects of antibiotics may involve disruption of the gut microbiome. Many studies have analyzed stool samples from COVID-19 patients and found significant changes in the composition of the gut microbiota [19], particularly severe depletion of bacterial species for potential immune modulation, which is also associated with COVID-19 Patients are associated with elevated levels of cytokines and inflammatory markers [20]. Therefore, these suggest that we should pay more attention to the time and type of antibiotic exposure and its adverse consequences in antibiotic stewardship to improve awareness of the appropriate use of antibiotics.

Additionally, previous studies have reported an association between higher NLR and increased risk of death in the general population [21, 22]. This may be due to the strong relationship between excessive inflammation and immunosuppression caused by SARS-CoV-2 infection [23, 24]. Recently, in COVID-19, multiple studies have also shown that NLR is associated with disease severity and mortality, and a meta-analysis concluded that assessing NLR can help clinicians detect severe COVID-19 cases early and initiate management promptly, thereby potentially reducing mortality [22]. We should also observe that NLR, as a cost-effective marker, can be easily calculated from routine peripheral blood tests, which provides great convenience to clinicians. Our study shows that the predictive performance of NLR remains applicable to CPAP patients during a pandemic of Omicron strains. NLR has also been previously proposed as a compass for predicting the efficacy of corticosteroid treatment in COVID-19 patients [25]. However, it is crucial to note that elevated levels of corticosteroids can increase neutrophil counts while simultaneously decreasing lymphocyte counts [26]. Therefore, it is imperative to recognize the need for future high-quality research that, considering various confounding factors, precisely elucidates the role of NLR in the progression and prognosis of COVID-19.

We acknowledge the limitations of this study, such as our data being a small cohort from a single center, and we should carefully consider the current research results. However, the small sample size is attributed to the nature of the study population, which focuses on individuals infected with the Omicron strain. At present, there is not enough strong evidence data about the new subvariants transmission, and our research can increase the understanding of the characteristics of CAPA after the invasion of the current Omicron strain. In addition, we recommend early identification of CAPA patients, early stratification of high-risk populations, and appropriate intervention measures.

### Electronic supplementary material

Below is the link to the electronic supplementary material.

**Supplementary Material 1: Supplementary Table 1.** Comparison of laboratory results of CAPA group and non-fungal infection group. **Supplementary Table 2.** Differences between survival group and death group in patients with CAPA

## Data Availability

The data used and/or analyzed during the current study are available from the corresponding author upon reasonable request.
