# Supplementary material for: Clinical courses and outcomes of COVID-19 associated pulmonary aspergillosis in 168 patients with the SARS-CoV-2 omicron variant
Source: BMC Infect Dis. 2024 Jan 23;24:117. doi: 10.1186/s12879-023-08971-w (PMC10804746; doi:10.1186/s12879-023-08971-w)
Supplement: Supplementary file 1 — Supplementary Material 1: Supplementary Table 1. Comparison of laboratory results of CAPA group and non-fungal infection group. Supplementary Table 2. Differences between survival group and death group in patients with CAPA [file 12879_2023_8971_MOESM1_ESM.docx]

**Supplementary table 1.** Comparison of laboratory results of CAPA group and non-fungal infection group.

| **Variables** | **On admission** | | | **Worst during hospitalization** | | |
| --- | --- | --- | --- | --- | --- | --- |
|  | **CAPA**  **(n=168)** | **Non-fungal infection (n=168)** | **P** | **CAPA**  **(n=168)** | **Non-fungal infection (n=168)** | **P** |
| SaO_2_ (95~98%) | 95.0 (91.8~98.0) | 97.0 (85.0~98.0) | **0.005** | 86.0 (60.0~92.0) | 96.0 (91.0~97.8) | **<0.001** |
| PaO_2_ (83~108 mmHg) | 76.5 (60.8~97.3) | 87.0 (72.0~103.0) | **0.004** | 50.0 (35.0~62.0) | 75.0 (58.0~94.0) | **<0.001** |
| Lactic acid (0.36~1.25 mmol/L) | 1.8±1.1 | 1.6±0.9 | **0.032** | 4.7±3.9 | 2.2±2.5 | **<0.001** |
| WBC count (3.5~9.5×10^9^/L) | 7.2 (4.7~11.0) | 5.5 (3.7~8.6) | **<0.001** | 14.9 (9.3~22.2) | 8.2 (5.6~11.8) | **<0.001** |
| Neutrophil count (1.8~6.3×10^9^/L) | 5.8 (3.3~9.6) | 4.1 (2.5~7.0) | **<0.001** | 13.2 (7.6~21.1) | 6.4 (4.0~9.8) | **<0.001** |
| Lymphocyte count (1.1~3.2×10^9^/L) | 0.8 (0.4~1.1) | 0.9 (0.6~1.3) | **0.005** | 0.3 (0.2~0.6) | 0.8 (0.5~1.2) | **<0.001** |
| Lymphocyte count <1.0×10^9^/L, n (%) | 118 (70.2) | 98 (58.3) | **0.030** | 145 (86.3) | 105 (62.5) | **<0.001** |
| NLR (0.78~3.53) | 7.7 (3.4~18.9) | 4.5 (2.6~8.4) | **<0.001** | 36.1 (12.0~82.6) | 7.1 (3.7~16.2) | **<0.001** |
| Hemoglobin (130~175 g/L) | 118.0±26.3 | 126.9±19.1 | **<0.001** | 85.5±27.1 | 116.0±23.9 | **<0.001** |
| Platelet count (125~350×10^9^/L) | 174.0 (138.0~227.0) | 205.0 (152.0~257.0) | **0.009** | 99.0 (43.0~174.0) | 174.0 (130.0~248.0) | **<0.001** |
| CRP (0~10 mg/L) | 51.2 (17.8~106.4) | 21.0 (3.6~59.5) | **<0.001** | 105.2 (50.3~178.7) | 27.9 (6.4~82.6) | **<0.001** |
| Albumin (40~55 g/L) | 34.4±5.4 | 38.2±5.8 | **<0.001** | 29.4±6.5 | 35.4±6.3 | **<0.001** |
| ALT (9~50 U/L) | 27.0 (15.0~40.0) | 22 (14.0~35.0) | **0.039** | 50.0 (28.0~122.0) | 30.0 (21.0~45.0) | **<0.001** |
| AST (15~40 U/L) | 34.0 (21.0~53.3) | 25.0 (18.0~35.0) | **<0.001** | 54.0 (36.0~166.0) | 57.0 (42.0~76.0) | 0.779 |
| ALP (45~125 U/L) | 69.0 (56.0~97.0) | 64.5 (53.8~81.3) | **0.016** | 91.0 (64.0~124.0) | 67.0 (55.0~83.0) | **<0.001** |
| Bilirubin (0~23 mmol/L) | 11.0 (7.6~15.6) | 9.8 (7.7~13.1) | 0.054 | 18.2 (12.0~27.8) | 10.5 (8.5~14.3) | **<0.001** |
| Potassium (3.5~5.3 mmol/L) | 4.1±0.7 | 4.0±0.5 | 0.105 | 4.1±1.8 | 3.9±0.7 | 0.137 |
| Sodium (137~147 mmol/L) | 139.0 (135.0~142.0) | 140.0 (137.0~142.1) | 0.083 | 139.0 (130.0~151.0) | 139.3 (136.7~141.7) | 0.623 |
| Urea (3.6~9.5 mmol/L) | 8.2 (5.6~13.1) | 5.6 (4.5~7.4) | **<0.001** | 18.1 (9.3~30.8) | 6.8 (4.9~9.6) | **<0.001** |
| Creatinine (57~111 μmol/L) | 82.0 (60.3~120.5) | 66.5 (54.0~83.0) | **<0.001** | 115 (67.8~253.5) | 69.5 (57.0~86.0) | **<0.001** |
| LDH (120~250 U/L) | 330.5 (242.0~436.0) | 226.0 (197.0~286.0) | **<0.001** | 375.0 (292.0~496.5) | 230.0 (197.5~298.0) | **<0.001** |
| D-dimer (0~0.55 mg/L) | 1.5 (0.7~5.0) | 0.7 (0.4~1.4) | **<0.001** | 8.3 (2.6~24.5) | 0.8 (0.4~2.8) | **<0.001** |
| PT (9~13 sec) | 12.3 (11.5~13.5) | 12.5 (11.5~13.1) | 0.685 | 14.4 (12.9~17.8) | 12.8 (12.0~13.5) | **<0.001** |
| Fibrinogen (2~4 g/dL) | 4.6±1.8 | 4.4±1.7 | 0.525 | 4.7±2.4 | 4.3±1.9 | 0.132 |
| APTT (25~31.3 sec) | 30.9 (27.4~33.5) | 28.5 (25.8~31.2) | **<0.001** | 38.4 (30.7~55.9) | 30.1 (27.0~34.5) | **<0.001** |
| PCT (<0.05 ng/mL) | 0.3 (0.1~1.1) | 0.06 (0.04~0.11) | **<0.001** | 2.1 (0.3~8.8) | 0.06 (0.04~0.20) | **<0.001** |
| CKMB (0~5 ng/mL) | 1.2 (0.5~2.4) | 0.5 (0.2~1.4) | **<0.001** | 2.2 (0.7~5.7) | 0.7 (0.2~1.7) | **<0.001** |
| Ultratroponin I (0~0.04 ng/mL) | 0.03 (0.01~0.08) | 0.006 (0.006~0.025) | **<0.001** | 0.15 (0.03~1.20) | 0.006 (0.006~0.037) | **<0.001** |
| MYO (0~110 ug/ml) | 115.2 (63.8~251.8) | 59.2 (33.0~120.3) | **<0.001** | 245.4 (82.0~1000.0) | 63.3 (34.7~145.7) | **<0.001** |
| NT-proBNP (0~125pg/mL) | 635.0 (215.6~1843.5) | 115.0 (41.0~354.3) | **<0.001** | 2402.0 (663.6~10731.5) | 183.5 (55.8~906.8) | **<0.001** |
| Positive serum BDG, n (%) * | 7/126 (5.6) | 3/134 (2.2) | **0.206** |  |  |  |
| Positive serum galactomannan, n (%) * | 5/126 (4.0) | 2/134 (1.5) | **0.269** |  |  |  |
| IL-2 (≤11.4 pg/mL) | 3.3±5.3 | 3.0±0.9 | 0.580 |  |  |  |
| IL-4 (≤12.9 pg/mL) | 4.9 (4.6~5.2) | 4.8 (4.6~5.4) | 0.666 |  |  |  |
| IL-6 (≤20.0 pg/mL) | 40.1 (14.0~139.7) | 12.0 (4.7~47.0) | **<0.001** |  |  |  |
| IL-10 (≤5.9 pg/mL) | 7.6 (5.3~12.5) | 5.9 (4.5~8.2) | **0.005** |  |  |  |
| TNF (≤5.5 pg/mL) | 3.2 (2.8~4.2) | 3.5 (3.1~5.1) | 0.050 |  |  |  |
| Interferon-γ (≤17.3 pg/mL) | 2.8 (2.2~3.9) | 3.1 (2.3~4.5) | 0.329 |  |  |  |
| IL-17 (≤20.6 pg/mL) | 2.6 (1.9~4.2) | 3.4 (1.9~5.0) | 0.132 |  |  |  |
| IgG (8.6~17.4 g/L) | 11.9±4.3 | 12.4±3.7 | 0.526 |  |  |  |
| IgM (0.3~2.2 g/L) | 0.8 (0.6~1.0) | 0.9 (0.6~1.4) | **0.049** |  |  |  |
| IgA (1~4.2 g/L) | 2.0 (1.4~2.6) | 2.6 (1.6~3.4) | **0.036** |  |  |  |
| IgE (0~100 g/L) | 87.1 (18.4~182.0) | 51.6 (22.8~145.8) | 0.629 |  |  |  |
| C3 (0.7~1.4 g/L) | 0.7±0.2 | 0.9±0.2 | **<0.001** |  |  |  |
| C4 (0.1~0.4 g/L) | 0.18 (0.14~0.24) | 0.21 (0.17~0.27) | **0.027** |  |  |  |
| CD3 count (723~2737 pcs/uL) | 263.5 (166.8~458.0) | 515.0 (253.0~710.0) | **0.001** |  |  |  |
| CD4 count (404~1612 pcs/uL) | 148.5 (75.5~256.8) | 245.0 (128.0~426.0) | **0.002** |  |  |  |
| CD8 count (220~1129 pcs/uL) | 98.5 (49.0~157.5) | 182.0 (62.0~311.5) | **0.002** |  |  |  |
| CD19 count (80~616 pcs/uL) | 101.3±94.7 | 135.6±105.5 | 0.052 |  |  |  |
| CD16+56 count (84~724 pcs/uL) | 72.5 (31.8~141.5) | 127.0 (71.0~193.5) | **0.002** |  |  |  |

*In case % were calculated for less than the maximal number of patients, data for some patients were missing and the actual denominator is displayed.

Bold values represent p < 0.05, indicating statistical significance.

**Abbreviations:** CAPA, COVID-19 associated pulmonary aspergillosis; SaO2, oxygen saturation; PO2, partial pressure of oxygen; WBC, white blood cell; NLR, neutrophil-lymphocyte ratio; CRP, C-reactive protein; ALT, alanine aminotransferase; AST, aspartate aminotransferase; ALP, alkaline phosphatase; LDH, lactate dehydrogenase; PT, prothrombin time; APTT, activated partial thromboplastin time; PCT, procalcitonin; CKMB, creatine kinase isoenzyme MB; MYO, myoglobin; NT-proBNP, N-terminal pro-B-type natriuretic peptide; BDG, β-1-3-D-glucan; IL, interleukin; TNF, tumor necrosis factor; IgG, immunoglobulin G; IgM, immunoglobulin M; IgA, immunoglobulin A; IgE, immunoglobulin E; C3, complement 3; C4, complement 4; CD, cluster of differentiation.

**Supplementary table 2.** Differences between survival group and death group in patients with CAPA.

| Variables | Survival group (n=95) | Death group (n=73) | P |
| --- | --- | --- | --- |
| Male, n (%) | 64 (67.4) | 60 (82.2) | **0.035** |
| Age, years | 74 (59~82) | 75 (64~83) | 0.631 |
| Smoking history, n (%) | 19 (20.0) | 21 (28.8) | 0.205 |
| History of alcohol consumption, n (%) | 9 (9.5) | 11 (15.1) | 0.338 |
| Length of hospital stay, day | 23 (15.0~35.5) | 17 (10~28) | **0.001** |
| Vital signs on admission |  |  |  |
| Systolic blood pressure, mmHg | 128 (114~140) | 132 (118~145) | 0.397 |
| Diastolic blood pressure, mmHg | 74 (65~81) | 75 (64~83) | 0.897 |
| Heart rate, beats/min | 86 (76~99) | 85 (76~98) | 0.695 |
| Respiratory rate, breaths/ min | 20 (18~20) | 20 (19~23) | **0.036** |
| Comorbidity, n (%) |  | | |
| Hypertension | 48 (50.5) | 43 (58.9) | 0.349 |
| Diabetes | 28 (29.5) | 18 (24.7) | 0.601 |
| Coronary artery disease | 22 (23.2) | 20 (27.4) | 0.591 |
| Chronic obstructive pulmonary disease | 11 (11.6) | 11 (15.1) | 0.645 |
| Cerebrovascular disease | 14 (14.7) | 10 (13.7) | 1.000 |
| Malignancy | 20 (21.1) | 10 (13.7) | 0.231 |
| Chronic renal insufficiency | 12 (12.6) | 9 (12.3) | 1.000 |
| Solid-organ transplant | 4 (4.2) | 8 (11.0) | 0.131 |
| HIV infection | 1 (1.1) | 1 (1.4) | 1.000 |
| Immunocompromised status | 21 (22.1) | 11 (15.1) | 0.322 |
| First symptoms |  | | |
| Fever (temperature ≥37.0℃), n (%) | 14 (14.7) | 14 (19.2) | 0.532 |
| Maximum temperature, ℃ | 36.5 (36.2~36.8) | 36.6 (36.3~36.8) | 0.328 |
| Cough, n (%) | 81 (85.3) | 54 (74.0) | 0.080 |
| Expectoration, n (%) | 73 (76.8) | 46 (63.0) | 0.060 |
| Chest tightness, n (%) | 41 (43.2) | 28 (38.3) | 0.635 |
| Shortness of breath, n (%) | 46 (48.4) | 34 (46.6) | 0.877 |
| Fatigue, n (%) | 50 (52.6) | 41 (56.2) | 0.755 |
| Myalgia, n (%) | 3 (3.2) | 2 (2.7) | 1.000 |
| Nasal congestion, runny nose, n (%) | 3 (3.2) | 4 (5.5) | 0.470 |
| Nausea, Vomiting, n (%) | 8 (8.4) | 5 (6.8) | 0.778 |
| Abdominal pain, diarrhea, n (%) | 11 (11.6) | 8 (10.9) | 1.000 |

Bolded terms represent subheadings. Bold values represent p < 0.05, indicating statistical significance.

**Abbreviations**: CAPA, COVID-19 associated pulmonary aspergillosis; HIV, human immunodeficiency virus.
